# A Computational Study of Cytotoxicity of Substituted Amides of Pyrazine- 2-carboxylic acids Using QSAR and DFT Based Molecular Surface Electrostatic Potential 

**Published:** 2013

**Authors:** Sharieh Hosseini, Majid Monajjemi, Elahe Rajaeian, Mohammad Haghgu, Aliakbar Salari, Mohammad Reza Gholami

**Affiliations:** a *Department of Chemistry, Science and Research Branch, Islamic Azad University, Tehran, Iran. *; b *Department of Chemistry, Arak Branch, Islamic Azad University, Arak, Iran.*; c *Department of Chemistry, Payame Noor University, Iran. *; d *Department of Chemistry, Shahre Rey Branch, Islamic Azad University,Tehran, Iran. *

**Keywords:** QSAR, Cytotoxicity, Antifungal activity, Molecular surface potential

## Abstract

Pyrazine derivatives are important class of compounds with diverse biological and cytotoxic activities and clinical applications. In this study, B3 p 86 / 6 – 31 ^+ +^ G * was used to compute and map the molecular surface electrostatic potentials of a group of substituted amides of pyrazine-2-carboxylic acids to identify common features related to their subsequent cytotoxicities. Several statistical properties including potentials extrema (V_s__,min_,V_s ,max_), the average of positive electrostatic potential on the surface (V_s_^+^), the average of V(r) over the surface (V_s_) and the Lowest Unoccupied Molecular Orbital (LUMO) and system cytotoxicities were computed. Statistically, the most significant correlation is a five -parameter equation with correlation coefficient, R² values of 0.922 and R²_adj_ = 0.879. The obtained models allowed us to reveal cytotoxic activity of substituted amides of Pyrazine2- carboxcylic acid.

## Introduction

Recent years have seen increased incidence of tuberculosis in both developing and industrialized countries, the widespread emergence of drug-resistant strains and a deadly synergy with the human immunodeficiency virus (HIV) (1). 

Pyrazine and its derivatives form an important class of compounds in natural flavours and complex organic molecules (2). Pyrazines are responsible for the flavour of foodstuffs as diverse as cooked meats, cheese, tea and coffee. The 2-methylpyrazine is used in flavours in food and tobacco. In addition, the 2-methylpyrazine is an insecticide, photo medicine and pigment and sensitizer. 

Pyrazineamide (PZA) is a nicotinamide analogue that has been used for almost 50 years as a first-line drug to treat tuberculosis (3). PZA is bactericidal to semi dormant mycobacteria and reduces total treatment time (4). Although the exact biochemical basis of PZA activity *in-vivo *is not known, under acidic conditions, it is thought to be a prodrug of pyrazinoic acid, a compound with antimycobacterial activity (5). The finding that PZA-resistant strains lose amidase (Pyrazinamidase or nicotinamidase) activity and the hypothesis that amidase is required to convert PZA to pyrazionic acid interacellularly led to the recent synthesis and study of various prodrugs of Pyrazionic acid (6). Various compounds possessing-NHCO-grouping, for example, substituted amides, acyl and thioacyl anilides, benzanilides, phenyl carbamates, etc were found to inhibit photosynthetic electron transport (7-10). 

Amides of 2-alkylpyridine-4-carboxilic (11-12) and 2-alkylsulfanyl-4 –pyridinecarboxilic (12, 13) acids inhibited the oxygen evolution rate in Chlorella vulgaris, and their inhibitory activity depends on the lipophilicity of the compounds.

One of the major perquisites for pharmacological screening and drug development is the prediction of absorption, *e.g*., transport of a molecule through cellular membranes, i.e. bioavailability. Most frequently, the drugs cross biological barriers by passive transport, which strongly depends on lipophilicity (14).

Martin Dolezal *et al. *(15) have reported the synthesis of a series of substituted amides prepared from some Pyrazine-2-carboxylic acids and alkylated anilines. Also, they studied the structure -activity relationships and determined the importance of increased lipophilicity for antimicrobacterial, antifungal and photosynthesis – inhibiting evolution of newly prepared Pyrazine – 2 – carboxylic acid amides.

In this study, we used IC_50_ values for the inhibition of the oxygen evolution rate in spinach chloroplasts by Amides from the mentioned paper.

It is known that the biological activity correlates greatly with the structures of pyrazine derivatives. In this respect Quantitative Structure-Activity Relationships (QSAR) has emerged as a promising tool to quantitatively understand the relationships between molecular structures and biological activities. QSAR studies have been successfully employed in modern chemistry and biochemistry. Recently it has been demonstrated that analogous to the conventional applications of QSAR modeling for the analysis of datasets of bioactive organic molecules, its application to modeling Manufactured Nano Particles (MNP) can be useful for predicting activity profiles of novel MNPs solely from their descriptors and designing safer nanomaterials with desired properties (16).

## Experimental

*Theory and computational details*

*Molecular surface electrostatic potential (MSEP)*

Molecular surface electrostatic potential (MSEP), which is created on the surface of a molecule by its nuclei and electrons, is a well-established guide to physical properties and molecular interactive behavior (17, 18).

Unlike many of the other quantities used now and earlier, as indexes of physicochemical behavior, the electrostatic potential V(r) is a real physical property, the one that can be determined experimentally by diffraction methods as well as computationally.

The electrostatic potential V(r) is created in the space around a molecule by its nuclei and the electrons are given rigorously by Equation (1) : 

Vr=∑ZARA-r-∫p(f)f-r df          (Equation 1)

Where Z_A_ is the charge on nucleus A, located at R_A_ and ρ(r΄) is the electronic density (19). The molecular surface was taken to be the 0.001 μ contour of ρ(r΄) as proposed by Bader *et al*. (20). The quantities characterizing the MSEP are as follows (21): 1-Vs, max and Vs, min are the most positive and negative values of V(r) on the molecular surface, respectively. 2- Π, is the average deviation on the molecular surface, defined by Equation (2):

$\pi =\frac{1}{n}\sum_{i=1}^{\pi }\left[V_{s}\left(\mathrm{ri}\right)-V_{s}\right]$          (Equation 2)

Where Vs is the average of V(r) over the surface.

3 - V_s_^+^ and V_s_^-^ are the average of positive and negative electrostatic potentials on the surface of the molecules, respectively.

4 - σ²_+_ and σ²_- _the positive and negative variances of V(r) over the molecules, respectively, which are included in the σ²_tot_,. The total variances of V(r) over the surface of molecules, according to Equation (3) :

$\sigma_{\mathrm{tot}}^{2}=\sigma_{+}^{2}+\sigma_{-}^{2}=\frac{1}{m}\sum_{j=1}^{m}\left[V_{R}^{+}\left\{r_{j}\right\}-V_{s}^{+}+\frac{1}{h}\sum_{R=1}^{h}V_{2}^{-}\right]$          (Equation 3)

5 - VB, the balance between the positive and negative surface potentials, is defined by Equation (4):

$V=\frac{\sigma_{+}^{2}\sigma_{-}^{2}}{{\left(\sigma_{\mathrm{tot}}^{2}\right)}^{2}}$           (Equation 4)

The multilinear correlation regression (MLR) method was used to obtain the optimum correlation. 

*Calculation methods*

**Figure 1:**
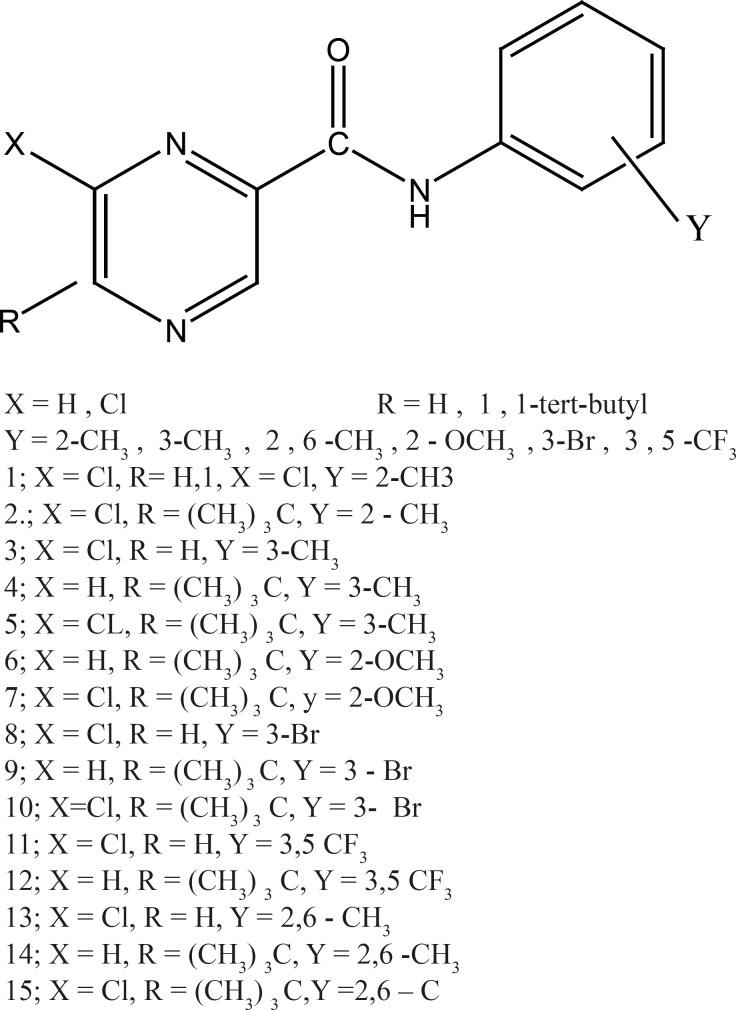
Structures of substituted Amides of Pyrazine-2-Carboxylic acids (1-15)

All the structures of Pyrazine amid 2-carboxylic acid derivatives are shown in Figure 1. The full geometry optimizations were performed by DFT method and 6-31^+ +^ G*basis set. All the calculations mentioned above were performed with the Gaussian 03 program package. 

*Molecular descriptors*

We derived some quantum descriptors from the DFT calculations, such as the Vs, max, 

V_s_, min, V_s_, V_s_^+^ and the Lowest Unoccupied Molecular Orbital (LUMO). 

*Stepwise multiple linear regression*

In order to select the predominant parameters that significantly affect the cytotoxicity of the compounds, we employed the statistic software SPSS, taking IC_50_ as the dependent variable and every candidate descriptor calculated above as an independent variable to perform the stepwise multiple linear regression. 

In the next step, QSAR equations were made through the multiple linear regression (MLR) method utilizing the five calculated descriptors. 

## Results and Discussion

*QSAR equation analysis and model validation*

The QSAR equation is demonstrated in Equation (5): IC_50_ = - 2.467 (±0.353 ) + 82.101 (±11.808) 1/ V_s,min_ - 34.882 (±4.031) LUMO - 0.132 (±0.036) < Vs > + 0.139 (±0.022 ) <V_s_^+^> + 5.569 (±2.416) 1/V_s,max_ (Equation 5) 

n =15, R²= 0.922, R²_adj_ = 0.879, SE = 0.095 

In which, n, S E and R² are the number of the compound analyzed, the correlation coefficient and the standard deviation respectively. 

**Figure 2:**
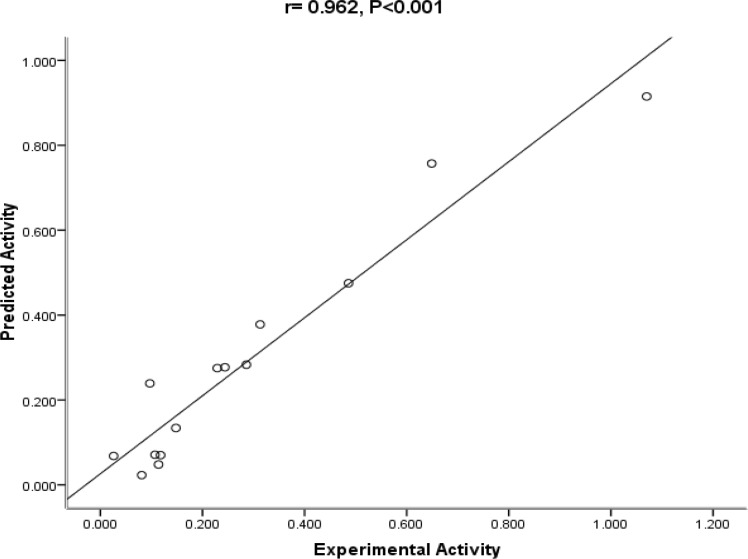
The Plot of predicted vs. experimental activity of substituted amides of Pyrazine - 2 – carboxylic acids

The mentioned indicators are usually used in QSAR analysis to judge how much the model is reliable. In order to check the reliability of the proposed equation, the observed versus predicted activities IC_50_ values according to the QSAR equation are plotted in Figure 2. As it can be seen, the experimental values are in good agreement with the predicted value, indicating the reliability of the equation. 

*Descriptores of the QSAR equation*

According to the equation, decreasing Vs, min and LUMO caused an increase in the drug activity and decreasing Vs, max could decrease the drug activity with lower speed. Hagelin *et al. *(22) showed an increase in Vs, min or Vs, max caused an increase in accepting and donating power of hydrogen bond, thus it could be predicted that an increase in these two quantities, interaction of drug molecule with solvent molecules will increase and lead to a decrease in the activity of the drug. The QSAR equation shows that the energy of the Lowest Unoccupied Molecular orbital (LUMO) affects the cytotoxity. The mentioned descriptor is an electronic parameter which directly relates to the electron affinity and characterizes the susceptibility of the molecule towards an attack by nucleophiles (23). 

The negative coefficient of the LUMO and Vs indicates that increasing their values can decrease the IC_50_. 

Vs, max is a parameter that is related to the solvent accessible surface of the compounds (24). The positive region of the surface electrostatic potentials of these molecules provides further contrasts. As mentioned above the strongest positive potentials, with Vs, max between 19.610 and 99.590 Kcal/ mol are produced by hydrogen of the amide group or ring hydrogens. However, there is no correlation between the number of available hydrogens and their molecules subsequent Vs, max, indicating that the positive region on their surfaces is relatively weak. On the other hand the negative surface region while less extensive in area, is much more uniform in strength. The Vs, min are all within a relatively narrow range, -24 to -47.140 Kcal/mol, which seems realistic to conclude that the negative potentials are of primary importance in cytotoxicity of amides. The results of our study was consistent with the finding of Fakhr (25). 

In considering those aspects, we can draw a conclusion that the cytotoxicites of the investigated compounds are influenced by both the structural and electronic properties. Therefore, the electronic and structural properties are important factors in the interaction between Pyrazin2-carboxylic acid derivatives that present cytotoxicity and the biological receptor.

In addition, the experimental results show that the compounds with 2-CH3 substituent on the phenyl ring (1, 2, 13, 14, and 15) had lower biological activity than the other investigated compounds. Consequently they assume that the methyl substituent in ortho position of the benzene ring is disadvantageous to the viewpoint of interactions with photosynthetic apparatus. The results of our study were consistent with the finding of Martin Dolezal *et al. *(15). 

Table 1 shows the experimentally determined and actual activity. Some of the key features of the molecular surface electrostatic potentials on the basis of our calculation are also listed in Table 1.

**Table 1:** Actual and predicted activity and molecular descriptors used in this study

**No.**	**Actual activity** **mmol/dm³**	**1/Vs, max** **mol/Kcal**	**1/Vs, min** **mol/Kcal**	**LUMO** **mol/Kcal**	**<Vs>** **mol/Kcal**	**<Vs+>** **mol/Kcal**	**Predicted activity** **mmol/dm³**
1	1.070	0.036231884058	-0.028793550245	-0.12697	1.770	9.71	0.916
2	0.244	0.050994390617	-0.030284675954	-0.11702	1.930	7.34	0.178
3	0.486	0.025419420437	-0.032164683178	-0.12618	2.070	9.45	0.475
4	0.148	0.031220730565	-0.027502750275	-0.10432	1.150	8.62	0.134
5	0.118	0.027233115468	-0.029744199881	-0.11628	1.980	7.43	0.070
6	0.286	0.038925652005	-0.026198585276	-0.09969	0.740	9.39	0.284
7	0.097	0.042589437819	-0.028546959749	-0.11178	1.080	7.60	0.239
8	0.313	0.023110700254	-0.036630036630	-0.13221	3.080	10.93	0.379
9	0.081	0.010163634516	-0.029655990510	-0.11040	1.640	8.88	0.023
10	0.107	0.010383137784	-0.028612303290	-0.12205	1.300	5.35	0.071
11	0.026	0.019364833462	-0.041666666667	-0.14175	5.890	12.10	0.069
12	0.114	0.020512820513	-0.034141345169	-0.12015	4.600	11.66	0.049
13	0.649	0.027685492802	-0.028288543140	-0.12480	1.650	9.05	0.758
14	0.229	0.010041168792	-0.021213406873	-0.10284	2.730	8.64	0.275
15	0.242	0.030571690614	-0.027056277056	-0.11469	1.310	7.02	0.285

 In seeking an analytical representation of the experimental data in Table 1, we tested a number of quantities related to Vs(r), including some which are shown in Table1. The best correlation was obtained by Equation 5. Although this equation does not reproduce the absolute values of the experimental data, it can predict the activity of the drug.

The above data was used to find a regression analysis of the correlation between the descriptors (Table 2). 

**Table 2:** Model Summary

**Model Summary**				
Model	R	R Square	Adjusted R Square	Std. Error of the Estimate
LUMO	0.232	0.054	-0.02	0.279
LUMO, 1/ Vs, min	0.675	0.455	0.364	0.218
LUMO, 1/Vs, min, <vs+>	0.797	0.635	0.536	0.186
LUMO, 1/ Vs, min, <vs+> , <Vs>	0.936	0.876	0.826	0.11
LUMO, 1/ Vs, min, <vs+>, <Vs> , 1/vsmax	0.96	0..922	0.879	0.095

## Conclusions

In this QSAR study, we have obtained an equation between descriptors and the cytotoxicity by combining the DFT theory method with statistical analysis. Since the electronic and structural descriptors are the main factors which influence the cytotoxicites of pyrazine 2 -carboxylic acid, it is necessary to explore such descriptors. Meanwhile, studying their applicability could lead to a vital improvement in QSAR.

The QSAR model could be helpful to estimate the activities of compounds by calculating the descriptors involved in the QSAR equation. 
